# Montelukast Improves Symptoms and Lung Function in Asthmatic Women Compared With Men

**DOI:** 10.3389/fphar.2019.01094

**Published:** 2019-09-24

**Authors:** Renata Esposito, Giuseppe Spaziano, Domenico Giannattasio, Francesco Ferrigno, Angela Liparulo, Antonietta Rossi, Fiorentina Roviezzo, Maurizio Sessa, Maddalena Falciani, Liberato Berrino, Mario Polverino, Francesca Polverino, Bruno D’Agostino

**Affiliations:** ^1^Department of Experimental Medicine, Section of Pharmacology “L. Donatelli”, University of Campania “L. Vanvitelli”, Naples, Italy; ^2^Pulmonary and Critical Care Medicine, Ospedale Scarlato, Scafati, Italy; ^3^Department of Pharmacy, School of Medicine, University of Naples Federico II, Naples, Italy; ^4^Department of Drug Design and Pharmacology, University of Copenhagen, Copenhagen, Denmark; ^5^Asthma and Airway Disease Research Center, University of Arizona, Tucson, AZ, United States

**Keywords:** montelukast, leukotrienes, eosinophils, asthma, nitric oxide, gender differences

## Abstract

**Purpose:** Gender differences exist in the prevalence of asthma and allergic diseases, partially due to the effects of sex hormones on the development of allergic manifestations. Women, compared with men, are more prone to suffer allergic asthma, experience difficulties in controlling asthma symptoms, and show adverse responses to drugs. However, there are knowledge gaps on the effectiveness of anti-leukotrienes drugs on lung function, symptoms, and pulmonary and systemic inflammation in adult asthmatic women compared with men. We conducted a prospective cohort study to characterize the effectiveness of an anti-leukotrienes drug, montelukast (MS), in asthmatic adult women and men.

**Methods:** Twenty-one asthmatic subjects (11 women and 10 men), who were on low-dose inhaled corticosteroids (ICS), were treated with MS. The optimal control of the symptoms was achieved in both groups according to the Global Initiative for Asthma guidelines. At enrollment, and after 13 weeks from the beginning of MS, pulmonary function tests and asthma control tests were performed, and the fraction of exhaled nitric oxide and blood eosinophils levels were measured.

**Results:** From baseline until the end of the study, women treated with MS + ICS had better control of the asthmatic symptoms, defined as higher asthma control test (ACT) score (17.00 ± 1.07 to 23.36 ± 0.45; p < 0.0015), improved pulmonary function [with higher forced expiratory volume in 1 s (from 77.25 ± 6.79 to 103.88 ± 6.24; p < 0.0077)], and forced vital capacity (from 91.95 ± 6.81 to 113.17 ± 4.79; p < 0.0183) compared with men. Interestingly, MS + ICS-treated women had significantly lower levels of blood eosinophils (from 5.27 ± 0.30 to 3.30 ± 0.31; p < 0.0449) and exhaled nitric oxide (from 44.70 ± 7.30 to 25.20 ± 3.90; p < 0.0294) compared with men.

**Conclusion:** The treatment with MS, added to ICS, in women leads to better control of symptoms, better management of lung function, and decreased inflammation levels compared with ICS + MS treatment in men.

## Introduction

Asthma, a highly prevalent chronic inflammatory disease of the airways, is characterized by a variable degree of airflow obstruction (D’Agostino et al., 2010) and bronchial hyperresponsiveness (Locksley, 2010; Roviezzo et al., 2016), caused by chronic exposition to allergens (Hamid and Tulic, 2009; Spaziano et al., 2015) or associated to other diseases such as gastroesophageal reflux (Gallelli et al., 2017; Rouget et al., 2004; D’Agostino et al., 2005). Chronic airway inflammation substantially contributes to airflow limitation, respiratory symptoms, and disease chronicity (Myers, 2015). Crucial players in the development of chronic airway inflammation in asthma are airway structural cells and immune cells infiltrating the lung (T cells and eosinophils) (Chaplin 2002; Singh et al., 2016), which are pivotal sources of inflammatory mediators such as leukotrienes (LT) (Filosa et al., 2015; Bruno et al., 2018). It is well recognized that women have a higher incidence of diseases with predominant LT activity, such as asthma and allergic rhinitis (Voskuhl, 2011). This higher incidence has been associated with biological susceptibility, age-related changes in the hormonal milieu, environmental exposures, as well as health care and socioeconomic factors (Barnes, 2011; Matteis et al., 2014). Asthmatic women experience worsening of the asthma symptoms during the premenstrual or menstrual phases of their cycle, suggesting that hormonal fluctuations during the menstrual cycle contribute to periodic worsening of the disease (Chen et al., 1994). Interestingly, epidemiological studies of both incidence and prevalence of asthma have reported a male predominance of asthma before puberty and a female predominance after puberty (Matteis et al., 2014). Recent evidence seems to suggest that such differences are due to gender differences in pulmonary LT levels in women versus men (Rossi et al., 2019). Such gender differences in LT synthesis may affect the greater severity of inflammatory responses in women compared with men. In fact, during inflammatory reactions, androgens influence 5-lipoxygenase (5-LO)-mediated cellular events (Sessa et al., 2018), inducing lower LT biosynthesis in men compared with women (Rossi et al., 2019). Based on this evidence, some study suggested that the gender could play a role on the effectiveness of anti-asthmatic pharmacological treatments, such as montelukast (MS). Currently, it is unknown whether gender differences could have an effect on the asthmatic symptoms, pulmonary function, and biomarkers associated with the severity of asthma [e.g., eosinophils and exhaled nitric oxide (FeNO)] (Sessa et al., 2018) in the adult population. MS is the most widely used cysteinyl leukotriene (cys-LT) receptor antagonist in Europe (Theron et al., 2014). It has both anti-inflammatory and bronchodilator activities, and it is widely used in the treatment of asthma. MS has been observed to be more efficient in the control of the asthma symptoms in girls reaching the puberty, compared with age-matched boys (Harmanci, 2007; Sessa et al., 2018). On the other hand, several studies (Pedersen et al., 1997; Biernacki et al., 2005; Burke et al., 2016) have shown that not all the asthmatic patients treated with MS experience a significant clinical improvement, and no factors have been identified to reliably predict the clinical response to cys-LT antagonists. Additionally, published clinical trials targeting adult populations did not examine gender subgroups separately, and thus, they could have underestimated the effects of the sex on the primary treatment outcomes. Because asthma is a complex disease, likely several factors, which have not been elucidated so far, contribute to the different response to therapy in women and men. Given the major role of LTs in the female biology, the gender differences should be taken in consideration when evaluating the therapeutic potential of anti-LT drugs as well as the efficacy of LT inhibitors in asthma.

To overcome these gaps in knowledge, this study aimed at evaluating the gender differences in asthmatic symptoms, pulmonary function, and biomarkers between men and women patients with asthma who were treated with MS + inhaled corticosteroids (ICS).

## Methods

### Study Participants

#### Recruitment

The patients were recruited from the outpatient clinics of the Pulmonary and Critical Care Medicine department of “Mauro Scarlato” Hospital in Scafati, Italy.

#### Characteristics of the Participants

Asthmatic patients with a history of atopy and documented presence of allergy (see later) were included. We excluded individuals with a history of lung diseases other than asthma, coronary artery disease, congestive heart failure, or any comorbidity such as gastroesophageal reflux or rhino-sinusitis, which could have had an effect on symptoms.

### Asthma Diagnosis

Asthma was diagnosed based upon the presence of both: a) respiratory symptoms (wheeze, shortness of breath, chest tightness, and cough) usually triggered by exercise, laughter, allergens, or cold air and worsening at night or on waking or exacerbated by viral infections; and b) expiratory airflow limitation. The asthmatic airflow phenotype was confirmed by one or more of the following: 1) increase in forced expiratory volume in 1 s (FEV_1.0_) of >12% and >200 ml from baseline in response to 400-mcg albuterol; or 2) excessive variability in twice-daily peak expiratory flow over 2 weeks (average daily diurnal peak expiratory flow variability >20%); or 3) positive bronchial challenge test (drop of ≥20% in FEV_1.0_ from baseline in response to standard doses of methacholine). The patients underwent intradermal skin testing for immunoglobulin E (IgE)-mediated hypersensitivity. The subjects did not use anti-histaminic drugs for at least 48 h before the skin testing. Allergy skin-prick tests were performed using 14 common aeroallergen extracts (Lofarma, Italy): *Dermatophagoides farinae*, *D. pteronyssinus*, cat, dog, ragweed mix, grass mix (timothy, june, orchard), ash, beech, birch, hickory, oak, poplar, and the molds *Aspergillus* and *Alternaria tenuis*; histamine (1 mg/ml) and saline (0.9%) solutions were used as positive and negative controls, respectively. Atopy was defined as a positive intradermal test to at least one allergen with a 2+ or greater wheal-and-flare response with or without pseudopods (21 to 30 mm of erythema with a 5- to 10-mm wheal). Histamine reactivity was documented by an intradermal injection of 0.05 ml of a solution containing histamine at 1 mg/ml. Serum samples were screened for total and specific IgE. Total IgE were evaluated by Fluoro-Fast Test (3M Diagnostics Inc., Santa Clara, CA), and an IgE level above 100 IU/ml was considered to be indicative of high probability of atopy. The diagnosis of asthma was successively confirmed by a positive radioallergosorbent (RAST) test for IgE antibodies (RAST-CAP-FEIA, Pharmacia, Uppsala, Sweden). The RAST results were classified as Class 1 (low level of specific IgE), Class 2 (moderate level), Class 3 (high level), and Class 4 (very high level). Only patients with both positive intradermal test and RAST were enrolled.

### Enrollment Procedures and Study Setting

Following enrollment, two women and two men were excluded from the analysis for no-show at the follow-up visit. The final study population included 10 males between 23 and 43 years old and 11 age-matched females; among the participants, there were two mild smokers (one male and one female) with a smoking history of 8.2 + 2.9 pack/years. No woman was taking oral or intrauterine contraceptives. Obesity, defined as a body mass index ≥30 kg/m^2^, was present (<35 kg/m^2^) in two females (**Table 1**). At the moment of the enrollment, all the subjects were treated using the Step 2 approach recommended by Consensus-based () Asthma Symptom Control (https://ginasthma.org/): regular low dose of ICS plus as-needed short acting beta2-agonists (SABA). Since the asthma was non- or partially controlled based upon daytime asthma symptoms, night waking due to asthma, as-needed use of SABA, activity limitation due to the symptoms, a step-up approach (Step 3, GINA guidelines) was required at baseline visit. According to the Step 3 approach, the subjects were switched to a low-dose maintenance therapy with ICS [100–200 mcg of beclomethasone dipropionate (HFA) per day] + MS (10 mg per day), plus as-needed SABA or low-dose ICS/LABA.

**Table 1:** Demographic characteristics of study participants.

Participant demographics	
Total participants (n)	21
Gender: Female (n)	11
Gender: Male(n)	10
Mean Age (years)	33 ± 10
Body mass index (kg/m^2^)	M: 25.8 ± 2.8 W: 25.2 ± 4.2
Current Smokers (n) (8.2 ± 2.9 pack/years)	1 man, 1 woman
Age at asthma onset (years), mean ± SD % with onset >12 years of age	45 ± 5 100 %
Exacerbations n/years	3

#### Baseline and End of Study Visits

At enrollment, prior to the MS addition to ICS therapy, the values for FEV_1.0_, FVC, fraction of FeNO, and eosinophils levels were recorded, together with the results of the asthma control test (ACT). Patients were seen by their assigned physician in an outpatient setting at enrollment and 13 weeks after the enrollment.

#### Assessments

Spirometry was performed according to the American Thoracic Society/European Respiratory Society guidelines. Spirometric maneuvers were conducted in triplicate, and the highest sum of FEV_1.0_ and FVC values was used in subsequent analyses. Predicted normal values of FEV_1.0_ and FVC were derived from standard equations.

The eosinophil count was performed by diluting whole blood with a phyloxine B staining solution that stains the eosinophil population in red. The diluted sample were charged onto a hemacytometer for counting using a low-power (10×) objective.

The fraction of FeNO was measured by using a portable electrochemical analyzer (NIOX VERO, by Vizient Inc, company, USA) according to the manufacturer’s instructions. All patients were instructed to exhale with a constant expiration flow for 10 s, and the plateau value of NO was recorded. Maneuvers with irregular tracings were rejected. Participants repeated the test until three acceptable tests were performed. To avoid any influence of forced expiration on the NO measurements, spirometry was performed after FeNO measurement. Each FeNO measurement was repeated three times by two physicians who were blinded to the clinical information, and then the average levels of FeNO were calculated.

The ACT is a five-point questionnaire assessing asthma symptoms (daytime and nocturnal), use of rescue medications, and the effect of asthma on daily activities. Each item includes five answers corresponding to a five-point Likert-type rating scale. At the end of the test, the answers for each of the five items are summed to yield a score ranging from 5 (poor control of asthma) to 25 (complete control of asthma).

The same parameters (FEV_1.0_, FVC, FeNO, circulating eosinophil number, and ACT test score) were assessed at the 13-week visit.

### Data Analysis

Differences in ACT score, FeNO levels, FEV_1.0_ and FVC values, and eosinophil counts between men and women in the pre- and posttreatment phases were analyzed by means of the Kruskal–Wallis test with the Mann–Whiney U test for *post hoc* analysis for data that were not normally distributed and by one-way analysis of variance, with Tukey post-test for pairwise comparisons for data that were normally distributed. For statistical computations, GraphPad InStat (GraphPad Software Inc., San Diego, CA) was used. The results were expressed as mean ± S.E.M. *P*-values < 0.05 were considered as significant.

#### Power Analysis

G-power 3.1.9.2 (Universität Kiel, Germany) was used for power analyses (Faul et al., 2009). We computed power given the effect size, the alpha error probability, and the total sample size. For the computation of the effect size, we used the Cohen’s d (Lakens, 2013).

### Ethics

The local Ethics Committee “ASL Salerno” approved the study (“GEAS” protocol, 2017 September 14^th^) and all respondents provided informed consent before participation.

## Results

### Gender Differences in Asthma Control Tests Among Patients Treated With Montelukast Plus Inhaled Corticosteroids

**Figure 1:**
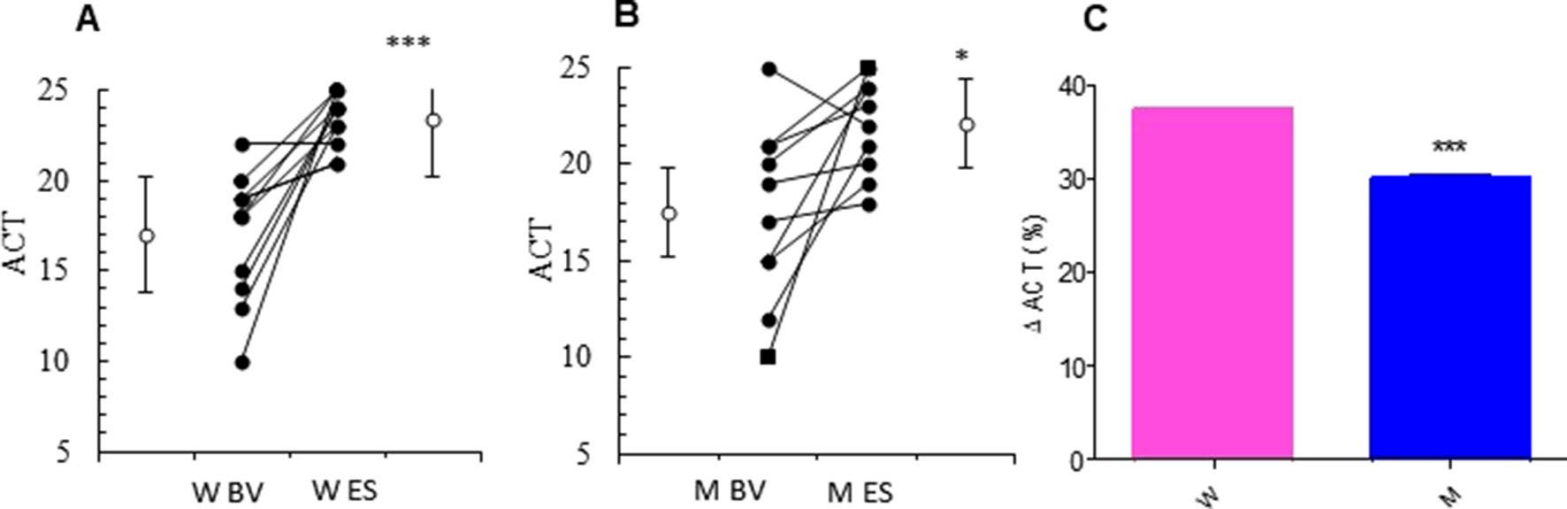
ACT evaluation. ACT evaluations in montelukast treated group in women **(A)** and in men **(B)** at the enrollment visit (EV) and at the end of study (ES). **(C)** Δ ACT (%) in treated women and in men. Results were shown as means ± SEM. The statistical tests used in these analyses were two-way analysis of variance followed by Student’s t-test. *P < 0.05; ***P < 0.001.

At baseline visit, there were no significant differences between women and men in ACT score. After 13 weeks of add-on therapy with MS, we observed a marked improvement in ACT score compared with the baseline one. The ACT score was significantly higher in women (from baseline 17.00 ± 1.07 to 23.36 ± 0.45; p < 0.0015) than men (from baseline 17.50 ± 1.44 to 22.10 ± 0.79; p < 0.0379) (**Figures 1A, B** and **Table 2**). Asthmatic women treated with MS + ICS showed a better control of symptoms compared with men treated with the same dose and therapeutic approach (**Figure 1C**). The higher effectiveness of MS + ICS in women vs. men is highlighted by the Delta Percentage (Δ%) (**Table 2**), which confirms the achievement of a better control of symptoms in women respect to men (p < 0.001).

**Table 2:** Data results of FEV_1.0_, FVC, FeNO, ACT, EOS, and Δ% indicated as mean ± SEM.

Stage	Treated (ICS + MS) Women					Treated (ICS + MS) Men				
	Pre-Treatment	Post-Treatment	Delta %			Pre-Treatment	Post-Treatment	Delta %		
	BV (mean ± SEM)	ES (mean ± SEM)	P value	(mean ± SEM)	P value	BV (mean ± SEM)	ES (mean ± SEM)	P value	(mean ± SEM)	P value
FEV_1.0_ %	77.25 ± 6.79	103.88 ± 6.24	0.008	22.84 ± 6.00	<0.001	78.88 ± 6.15	85.88 ± 6.14	NS	8.97 ± 6.00	<0.001
FVC %	91.95 ± 6.81	113.17 ± 4.79	0.018	22.80 ± 6.00	<0.001	81.11 ± 6.98	91.52 ± 6.53	NS	13.00 ± 4.00	<0.001
FeNO ppb	41.72 ± 4.26	18.18 ± 1.92	<0.001	–56.42 ± 5.00	<0.001	44.70 ± 7.30	25.20 ± 3.90	0.029	–43.60 ± 6.00	<0.001
ACT	17.00 ± 1.07	23.36 ± 0.45	0.001	37.41 ± 1.00	<0.001	17.50 ± 1.44	22.10 ± 0.79	0.038	30.00 ± 1.50	<0.001
Blood EOS %	5.27 ± 0.30	3.30 ± 0.31	0.045	–37.38 ± 0.30	<0.001	3.61 ± 0.70	3.19 ± 0.66	NS	–11.63 ± 0.60	<0.001

BV, baseline visit; ES, end of study visit; ppb; part per billion; EOS: eosinophils.

### Gender Differences in Lung Functions Among Patients Treated With Montelukast Plus Inhaled Corticosteroids

**Figure 2:**
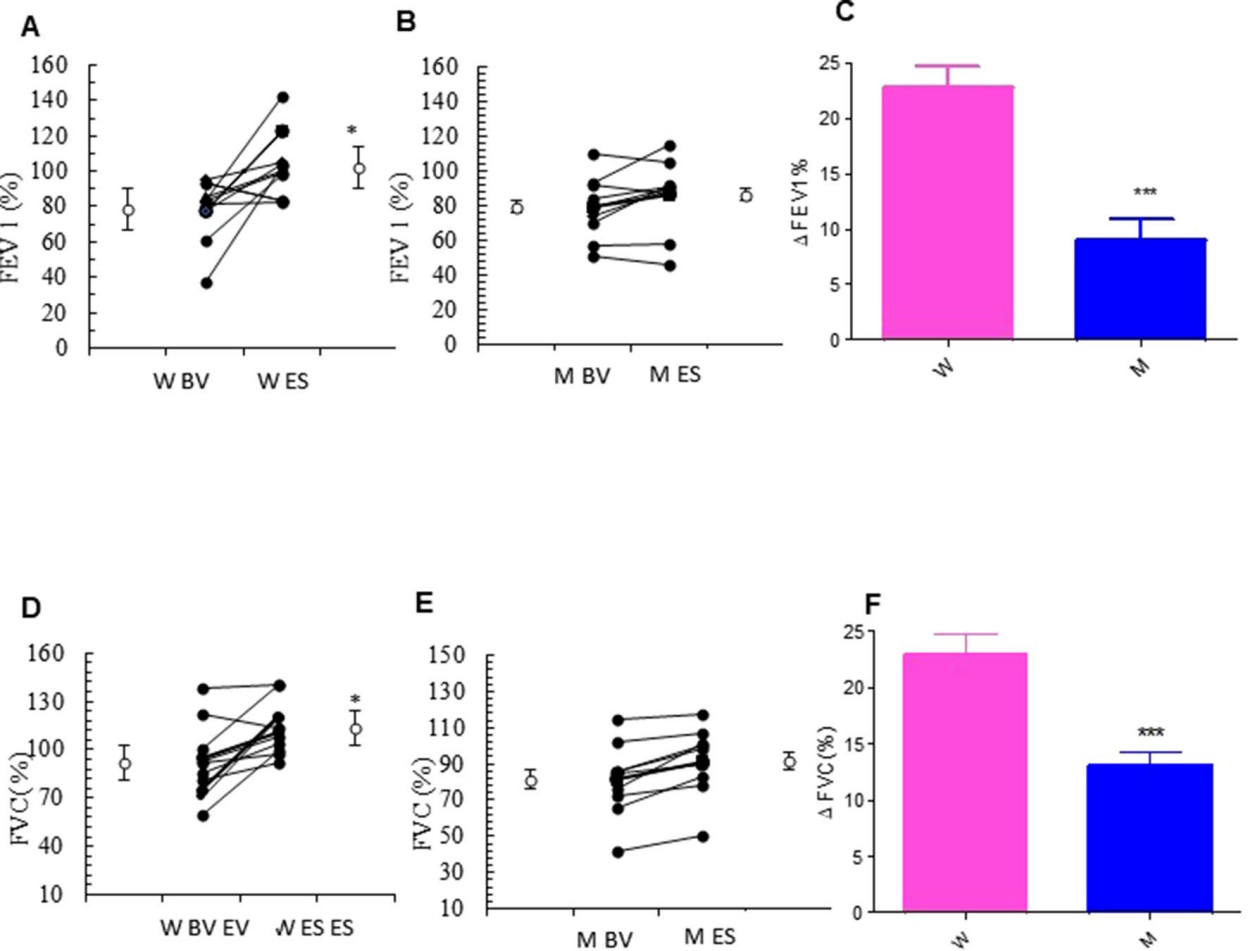
Forced expiratory volume in the 1st second (FEV_1.0_) and forced vital capacity (FVC) evaluation. FEV_1.0_ (%) in montelukast treated in women **(A)** and in men **(B)** at the enrollment visit (EV) and at the end of study (ES). **(C)** Δ FEV_1.0_ (%) in treated women and in men. FVC (%) in montelukast treated group in women **(D)** and in men **(E)** at EV and at ES. **(F)** Δ FVC (%) in treated women and in men. Results were shown as means ± SEM. The statistical tests used in these analyses were two-way analysis of variance followed by Student’s t-test. *P < 0.05; ***P < 0.001.

The addition of MS to ICS significantly and persistently enhanced FEV_1.0_ in asthmatic women, when compared with baseline FEV_1.0_ (from 77.25 ± 6.79% to 103.88 ± 6.24% p < 0.0077). In contrast, there were no differences in the values of FEV_1.0_ between baseline and follow-up visit in the asthmatic men group (**Figures 2A, B** and **Table 2**). Moreover, asthmatic women had a significantly better mean FVC at end of the study compared with FVC measured at baseline (from 91.95 ± 6.81% to 113.17 ± 4.79% p < 0.0183). This improvement was not observed in the men (**Figures 2D, E** and **Table 2**). These results are further confirmed by the evaluation of the Delta Percentage (Δ%) (**Figures 2C, F** and **Table 2**).

### Gender Differences in Inflammation Biomarkers Among Patients Treated With Montelukast Plus Inhaled Corticosteroids

**Figure 3:**
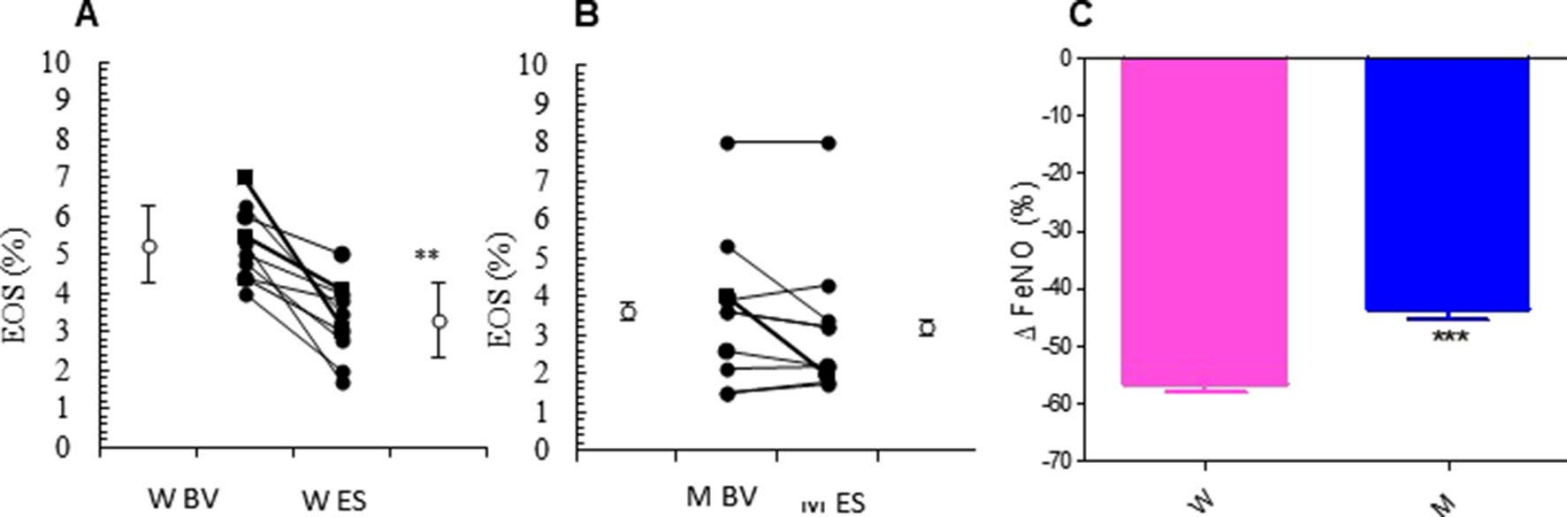
Eosinophils (EOS) levels. EOS (%) in montelukast treated group in women **(A)** and in men **(B)** at the enrollment visit (EV) and at the end of study (ES). **(C)** Δ EOS (%) in treated women and in men. Results were shown as means ± SEM. The statistical tests used in these analyses were two-way analysis of variance followed by Student’s t-test. **P < 0.01; ***P < 0.001.

**Figure 4:**
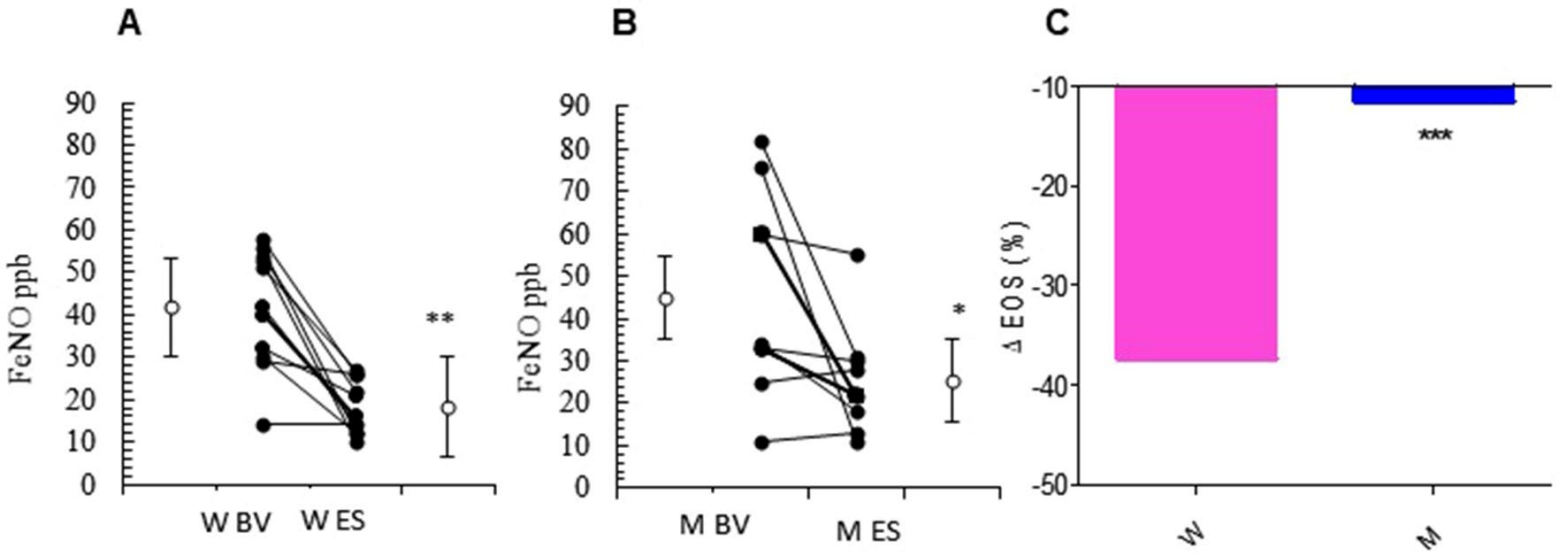
FeNO levels. FeNO level in montelukast treated group in women **(A)** and in men **(B)** at the enrollment visit (EV) and at the end of study (ES). **(C)** Δ FeNO (%) in treated women and in men. Results were shown as means ± SEM. The statistical tests used in these analyses were two-way analysis of variance followed by Student’s t-test. *P < 0.05; **P < 0.01; ***P < 0.001.

The relevant clinical and functional improvements were associated with reduction in blood eosinophils counts in the MS + ICS female group, where a significant decrease in blood eosinophil counts with respect to baseline (form 5.27 ± 0.30 to 3.30 ± 0.31; p < 0.0449, **Figures 3A, B** and **Table 2**) was observed. In asthmatic men, MS + ICS did not affect the blood eosinophil counts compared with baseline. Moreover, the Δ eosinophil% was significantly higher in women respect to men (p < 0.001) (**Figure 3C** and **Table 2**). The FeNO levels were also measured. FeNO reflects the airway inflammation, as it is mainly produced by inducible nitric oxide synthase, expressed by both bronchial epithelial and some inflammatory cells of asthmatic patients such as alveolar macrophages, neutrophils, and dentritic cells (Sandrini et al., 2003). At the final visit, the FeNO concentration in asthmatic women was significantly lower than the concentration at baseline (baseline: 41.72 ± 4.26 ppb; final visit: 18.18 ± 1.92 ppb; p < 0.001). Men treated with MS + ICS showed smaller differences in FeNO levels than women at baseline vs. 13 weeks visit (baseline: 44.70 ± 7.30 ppb; last visit: 25.20 ± 3.90 ppb; p < 0.0294, **Figures 4A, B** and **Table 2**). Furthermore, the FeNO Δ Percentage in men was significantly lower compared with women (**Figure 4C** and **Table 2**).

### Power Analyses

Results of power analysis are shown in **Table 1** of **Supplementary Material**.

## Discussion

This is the first prospective cohort study that documents a higher effectiveness of MS treatment on lung function, symptoms, and readouts of inflammation in asthmatic women compared with men, suggesting a sex-dependent effect of the treatment with MS in asthma.

Gender affects a wide range of biological functions. The physiology and the psychology of men and women are greatly different, and this diversity has a profound impact on the development, diagnosis, and treatment of any disease (Zein and Erzurum, 2015). Several studies showed that gender affects the onset and the progression of asthma, with male children having almost double risk for developing asthma with respect to age-matched girls. However, during adolescence, this prevalence equalizes, and by adulthood, asthma tends to be more prevalent in women (Pignataro et al., 2017). The goal of the pharmacologic treatment in asthma is to obtain and maintain a good control of symptoms (Gallelli et al., 2017). The ACT is a widely used questionnaire to keep track of the management of asthma symptoms from a patient’s perspective (Schatz et al., 2006). In our study, the treatment with MS, added to a baseline ICS therapy, improved the asthmatic symptoms in women significantly more than in men. The ACT results were supported by respiratory function evaluations. In fact, both FEV_1.0_ and FVC were significantly improved in asthmatic women respect to men at the end of the 13 weeks of MS + ICS treatment vs. baseline. Previous studies suggest that specific asthma phenotypes, such as exercise-induced asthma (Meltzer et al., 1996), asthma associated with allergic rhinitis or obesity (D’Agostino et al., 2002), asthma in smokers, asthma induced by aspirin (Israel et al., 1993; Roviezzo et al., 2015; Thomson, 2017), and asthma with predominantly small airway involvement (Tartaglione et al., 2018), are more likely to respond to LT receptor antagonists than others (Paggiaro and Bacci, 2011; Marcello and Carlo, 2016). The increased efficacy of anti-LT in association with ICS in improving the asthmatic symptoms compared with LABA + ICS has been linked to a constitutive overproduction of cys-LTs in the asthmatic phenotype. Therefore cys-LTs could have a major pathophysiological role in asthmatic women pathogenesis (Paggiaro and Bacci, 2011; Marcello and Carlo, 2016).

Gender has emerged as a key variable in the regulation of the 5-LO pathway and therefore in LT production. This links gender differences in LT production to the development of inflammatory responses (Rossi et al., 2014; Pace et al., 2017). In several animal models of acute inflammation, significantly higher cys-LT (LTC4 and LTB4) levels have been detected in the inflamed peritoneum of female mice and not in males (Pergola et al., 2008; Pergola et al., 2011; Rossi et al., 2014; Pace et al., 2017). Furthermore, in *ex vivo* studies on human neutrophils (the main source of LT in the blood), a higher production of LTs has been demonstrated in women. In particular, the levels of LTs were inversely correlated with the levels of testosterone, and 5α-dihydrotestosterone repressed LT synthesis in women down to the levels observed in men. Moreover, during inflammatory reactions, androgens orchestrate the events preceding the LT biosinthesis, such as 5-LO subcellular localization, phospholipase D activity, and the 5-LO/FLAP complex assembly. As a result, there is a lower LT biosynthesis in men versus women (Pergola et al., 2008; Pergola et al., 2011; Rossi et al., 2014; Pace et al., 2017). Eosinophils play an important role in the inflammation in asthma. In fact, their peripheral blood levels are significantly higher during acute asthma exacerbations compared with clinical remission (Fireman et al., 2011). LTs, and in particular cys-LTs, C4, D4, and E4, play key roles in airway eosinophilic inflammation in patients with asthma (Busse 1998; Drazen et al., 1999; Minoguchi et al., 2002). Interestingly, in our study, MS + ICS treatment reduced eosinophil levels from baseline vs. 13 weeks in both genders, but the decrease in eosinophil levels was statistically significant only in the female group. Several studies of asthmatic patients addressing the inflammatory patterns in response to anti-LT treatment led to contrasting results (Ye et al., 2015; Columbo 2017). Some studies, conducted in adult patients with persistent asthma, showed a significant improvement in respiratory function, asthma symptoms, and blood and sputum eosinophil counts, in response to the treatment with MS (Pizzichini et al., 1997; Minoguchi et al., 2002). In contrast, another study conducted on 25 elderly asthmatic subjects showed no significant effect of MS on the asthma symptoms and the levels of sputum and peripheral blood eosinophils levels (Columbo, 2017). However, in these studies, the patients were not divided according to their gender, and thus, it was not possible to show any difference in treatment efficacy between women and men (Ye et al., 2015; Columbo, 2017). In our study, MS + ICS treatment reduced FeNO levels in women significantly more than that in men. Previous studies have shown that MS treatment reduces FeNO levels in asthmatic children and adult (Sandrini et al., 2003; Straub et al., 2005), and no effects of inhaled fluticasone were observed in reducing the levels of FeNO (Fritscher et al., 2009; Rabinovitch et al., 2010).

### Limitations

Our findings should be interpreted in virtue of a set of limitations. First, only patients undergoing a step 3 therapy (MS + low ICS doses) for asthma were included in the study, and thus, the effects of MS in other stages of asthma cannot be extrapolated. Second, we have only evaluated blood peripheal eosinophils. The evaluation of lung eosinophils could have been more informative of the levels of lung inflammation. However, bronchoalveolar lavage fluid analysis was not allowed in our study population for etichal reasons. Third, the sample size is limited due to the exclusion criteria we applied in order to select a population of age- and gender-matched asthmatic people without comorbidities that would have biased the interpretation of the asthmatic symptoms and clinical outcomes. On the other hand, this choice may have reduced the generalizability of results. Fourth, the limitations related to the observational nature of this study should be considered in the interpretation of the results. Last, we cannot exclude that the effects observed after the step-up therapeutic approach was initiated are due to the synergistic effect of MS + ICS. However, as no differences between females and males were observed with the ICS therapy alone (Dunn et al., 2015), it is very likely that the differences observed when the subjects were assigned to MS + ICS therapy are mainly due to the addition of MS.

### Conclusion

Our results indicate that treatment with MS + ICS improves control of the asthma symptoms, lung function, and inflammation in women much more than in men. Future clinical studies are needed to clarify the beneficial effects of anti-LT in asthmatic women and men in order to set up a gender-tailored therapeutic approach to the disease.

## Data Availability

The datasets generated for this study are available on request to the corresponding author.

## Ethics Statement

The studies involving human participants were reviewed and approved by Ethics Committee “ASL Salerno.” The patients/participants provided their written informed consent to participate in this study.

## Author Contributions

Conceptualization: BD’A, RE, and MP. Data curation: GS, AL, and MS. Formal analysis: GS, AL, and MS. Funding acquisition: BD’A. Investigation: DG, FF, and MF. Methodology: AR and FR. Supervision: BD’A, MP, and FP. Validation: FP. Roles/writing—original draft: RE and GS. Writing—review and editing: BD’A, LB, and FP. All authors have approved the final article.

## Funding

This work was supported by PRIN 2015 no. 201532AHAE_004 from the Italian Ministry of Education, University and Research. MS’s postdoc is supported by a grant from the Novo Nordisk Foundation to the University of Copenhagen (NNF15SA0018404).

## Conflict of Interest Statement

The authors declare that the research was conducted in the absence of any commercial or financial relationships that could be construed as a potential conflict of interest.
