# Supplementary material for: Montelukast Improves Symptoms and Lung Function in Asthmatic Women Compared With Men
Source: Front Pharmacol. 2019 Sep 24;10:1094. doi: 10.3389/fphar.2019.01094 (PMC6769077; doi:10.3389/fphar.2019.01094)
Supplement: Supplementary file 1 [file Table_1.docx]

**Montelukast improves symptoms and lung function in women compared with men.**

***Gender-Specific Asthma Treatment with montelukast***

Renata Esposito^1^, Giuseppe Spaziano^1*^, Domenico Giannattasio^2^, Francesco Ferrigno^2^, Angela Liparulo^1^, Antonietta Rossi^4^, Fiorentina Roviezzo^4^, Maurizio Sessa^1,5^, Maddalena Falciani^2^, Liberato Berrino^1^, Mario Polverino^2^, Francesca Polverino^3^ and Bruno D'Agostino^1^

^1^Department of Experimental Medicine, Section of Pharmacology “L. Donatelli”, University of Campania “L. Vanvitelli”, Naples (Italy)

^2^ Pulmonary and Crirtical Care Medicine, Ospedale Scarlato, Scafati, - Scafati (Italy)

^3^ Asthma and Airway Disease Research Center, University of Arizona, Medicine, Tucson, Arizona, United States - Tucson (USA)

^4^ Department of Pharmacy, School of Medicine, University of Naples Federico II, Naples (Italy)

^5^ Department of Drug Design and Pharmacology, University of Copenhagen, Copenhagen, (Denmark)

**Corresponding author:**

^*^Giuseppe Spaziano, MSc, PhD

Department of Experimental Medicine, Section of Pharmacology “L. Donatelli”, University of Campania “L. Vanvitelli”, Via S.M. di Costantinopoli, 16, 80138 Naples, Italy

+39.0815667533

giuseppe.spaziano@unicampania.it

**Journal repository**

**Supplementary Table 1.** Post-hoc power analyses.

| **POST- Vs PRE-TREATMENT** | | | | | | **POST-TREATMENT: MEN VS WOMEN** | | | | | |
| --- | --- | --- | --- | --- | --- | --- | --- | --- | --- | --- | --- |
| **Analysis** | **Effect size (Cohen’s d)** | **Noncentrality parameter λ** | **Critical F** | **Denominator Degree of Freedom** | **Power (1-β error)** | **Analysis** | **Effect size (Cohen’s d)** | **Noncentrality parameter δ** | **Critical T** | **Degree of Freedom** | **Power (1-β error)** |
|  | **WOMEN** | | | | |  |  |  |  |  |  |
| **FEV1 %^⸸^** | 4.08 | 166.46 | 4.96 | 10.00 | 1.00 | **FEV1 %**^¥^ | 2.90 | 6.64 | 2.09 | 19 | 0.99 |
| **FVC %^⸸^** | 3.60 | 129.6 | 4.96 | 10.00 | 1.00 | **FVC %**^¥^ | 3.78 | 8.65 | 2.09 | 19 | 1.00 |
| **FeNO ppb^⸸^** | 7.12 | 506.94 | 4.96 | 10.00 | 1.00 | **FeNO ppb**^¥^ | 2.28 | 5.22 | 2.09 | 19 | 0.99 |
| **ACT^⸸^** | 7.75 | 600.62 | 4.96 | 10.00 | 1.00 | **ACT**^¥^ | 1.96 | 4.48 | 2.09 | 19 | 0.99 |
| **Blood EOS %^⸸^** | 6.46 | 417.08 | 4.96 | 10.00 | 1.00 | **Blood EOS %**^¥^ | 0.21 | 0.48 | 2.09 | 19 | 0.07 |
|  | **MEN** | | | | |  |  |  |  |  |  |
| **FEV1 %^⸸^** | 1.14 | 11.69 | 5.12 | 9.00 | 0.86 |  |  |  |  |  |  |
| **FVC %^⸸^** | 1.54 | 21.34 | 5.12 | 9.00 | 0.98 |  |  |  |  |  |  |
| **FeNO ppb^⸸^** | 3.33 | 99.80 | 5.12 | 9.00 | 1.00 |  |  |  |  |  |  |
| **ACT^⸸^** | 3.96 | 141.12 | 5.12 | 9.00 | 1.00 |  |  |  |  |  |  |
| **Blood EOS %^⸸^** | 0.06 | 0.03 | 5.12 | 9.00 | 0.05 |  |  |  |  |  |  |

**^⸸^** repeated measure ANOVA; ^¥^T-test (two tails).
